# Risk factors for fall occurrence in hospitalized adult patients: a case-control study^1^

**DOI:** 10.1590/1518-8345.2460.3016

**Published:** 2018-08-09

**Authors:** Isis Marques Severo, Ricardo de Souza Kuchenbecker, Débora Feijó Villas Boas Vieira, Amália de Fátima Lucena, Miriam de Abreu Almeida

**Affiliations:** 2 PhD, RN, Serviço de Terapia Intensiva, Hospital de Clínicas de Porto Alegre, Porto Alegre, RS, Brazil.; 3 PhD, Adjunct Professor, Faculdade de Medicina, Universidade Federal do Rio Grande do Sul, Porto Alegre, RS, Brazil.; 4 PhD, Adjunct Professor, Escola de Enfermagem, Universidade Federal do Rio Grande do Sul, Porto Alegre, RS, Brazil.

**Keywords:** Accidents by Falls, Risk Factors, Adult, Hospitals, Advanced Practice Nursing, Quantitative Analysis

## Abstract

**Objective:**

to identify risk factors for falls in hospitalized adult patients.

**Methods:**

a matched case-control study (one control for each case). A quantitative study conducted in clinical and surgical units of a teaching hospital in Southern Brazil. The sample comprised 358 patients. Data were collected over 18 months between 2013-2014. Data analysis was performed with descriptive statistics and conditional logistic regression using Microsoft Excel and SPSS version 18.0.

**Results:**

risk factors identified were: disorientation/confusion [OR 4.25 (1.99 to 9.08), p<0.001]; frequent urination [OR 4.50 (1.86 to 10.87), p=0.001]; walking limitation [OR 4.34 (2.05 to 9.14), p<0.001]; absence of caregiver [OR 0.37 (0.22 to 0.63), p<0.001]; postoperative period [OR 0.50 (0.26 to 0.94), p=0.03]; and number of medications administered within 72 hours prior the fall [OR 1.20 (1.04 to 1.39) p=0.01].

**Conclusion:**

risk for falls is multifactorial. However, understanding these factors provides support to clinical decision-making and positively influences patient safety.

## Introduction

According to the World Health Organization (WHO), fall is defined as “inadvertently coming to rest on the ground, floor or other lower level, excluding intentional change in position to rest in furniture, wall or other objects”^1^.

In hospitalized patients, incidence rates of falls are responsible for two in five adverse events, and their frequency varies from 1.3 to 13.0 per 1,000 patients per day^1^ ^-^ ^2^.

A recent study showed that in the United States of America (USA) the prevalence of falls increased from 28.2% to 36.3% in 2010^3^. In England and Wales, between 2008 and 2009, there were 283,438 notifications of the event^2^, and in Holland the number of admissions due to falls increased from 87.7 to 141.2 per 10,000 people in the period between 1981 and 2008^4^. In Austria, of the 3,648 patients investigated in hospitals, 38.5% suffered injuries due to falls. Similar results were found in Switzerland, where of the 10,098 patients, the prevalence of falls reached 34.7%^5^. This can be a result, possibly, of an increase in the number of notifications due to the aggravations that have occurred

The event can bring several consequences to patients, such as fractures, unanticipated vascular and indwelling catheters and drains removal, fear of falling, change of emotional status, clinical worsening, and even death. In addition to mortality, falls could increase the length of hospital stay and treatment costs^2^ ^,^ ^6^.

Falls and fall prevention have become an important theme across hospitals and other health care facilities, as well as across different countries. Regardless of geographic location, fall etiology is multifactorial, and its risk factors can be classified as intrinsic (patient-related) and/or extrinsic (environment and work-related).

Observational studies investigating these risk factors in hospitalized patients presented some possible bias, such as sample constituted exclusively of patients aged 65 or more^7^ ^-^ ^10^; investigation of events just within the first week of admission^8^; definition as exclusion criteria: patients with dementia, delirium or memory change^11^; and absence of data collection on Sundays and Holidays^7^.

In this context, the hypothesis of this study was that the identification of risk factors for falls in hospitalized adult patients facilitates a more accurate measurement of the risk for falls, and has a positive impact on patient safety. Therefore, the objective of this study was to identify predictors for falls in hospitalized adult patients.

## Method

This is an observational case-control study (one control for each case) with matching. Patients were matched regarding sex, unit and date of admission. The outcome was the incidence or not of fall(s). First, patients who have suffered falls were selected (cases). Next, subjects who have not suffered falls were selected (controls).

The study setting comprised 12 clinical and surgical units of an 843-beds hospital, connected academically to an university in Southern Brazil, which was recently accredited by the Joint Commission International (JCI)^12^. In this institution, nurses report hospital inpatient falls in the electronic health record. This notification creates an e-mail that is sent to the multi-professional team responsible for risk management and patient safety. During the study data collection, the investigators received this same e-mail and conducted an active search in the units during all weekdays, covering all shifts, in order to identify the incidence of falls.

The sample consisted of 358 clinical and surgical patients. Patients included were 18 years old or older, both sexes, controls with the same admission date as the cases, or subsequent dates. Exclusion criteria were: patients without clinical conditions (torpor or coma) to participate in the study, those who did not have caregiver in the time of data collection, patients under palliative care, those whose falls occurred outside the units of study and those whose falls occurred for the second time (or more). 

The study protocol specified no more than 72 hours after the fall for including patients in the study.

Data were collected over 18 months, between 2013-2014, by the researcher, four registered nurses and one Nursing student, and they received specific training before data collection. The training comprised theoretical (three-hour-long meetings) and theoretical-practical classes (daily supervision by the principal investigator on the logistic of the research assistants, and in the field, between April and July 2013). The evaluation of the event, data collection technique and documentation were carried out in conjunction. After these three months, the research assistants were considered capable of collecting the data individually.

Data were collected directly from patients, from the electronic health record, from the fall risk scale adopted in the hospital (Morse Fall Scale)^13^ ^-^ ^14^, and from the institutional instrument for describing falls. This instrument is composed of the factors that trigger the fall and patient clinical conditions before the event.

**Figure 1:**
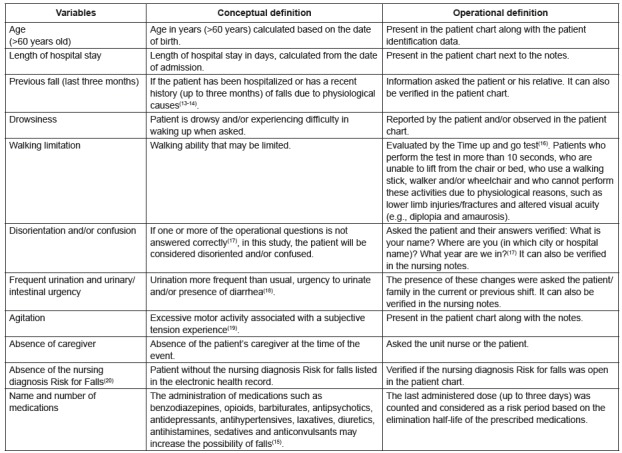
Conceptual and operational definitions of the study variables. Porto Alegre, RS, Brazil, 2013-2014

The variables (risk factors) of the study were selected from a previous study^15^ ^)^ and included in a data collection manual. Conceptual and operational definitions were constructed for the included variables (Figure 1): 

The collected data were double entered into Microsoft Excel. The statistical analysis was performed using Excel (Microsoft Office 2013) version 15.0 and SPSS (Statistical Analysis System, Chicago, EUA) version 18.0. 

The sample was paired using SPSS 18.0. The continuous variables with normal distribution were represented as mean, standard deviation and a 95% confidence interval (CI); and the asymmetric variables were represented as median and interquartile range. Normal distribution was evaluated using histograms. Categorical variables were represented as percentages and absolute numbers.

The relationship between the outcome and the predictive variables was analyzed by conditional logistic regression. The variables with p-value <0.25, 95%CI higher <8.0 and/or lower >0.025 were included in the univariate logistic regression, and their ordering was performed by 2log likelihood values. Next, a multivariate logistic regression with a backward elimination was carried out until variables with a p*-*value <0.05 and/or with clinical/scientific significance remained, independently of the p-value. 

The sample calculation was performed according to Chang and et al^9^, from the therapy of narcotics, with odds ratio (OR)=2.13 and a prevalence of falls of 13.9%. It was considered a statistical power of 80% and a significance level of 0.05, with 20% of possible losses that could occur during the study. 

The Research Ethics Committee of the hospital approved this study (protocol #130012).

## Results

The sample consisted of 54% (n=204) of male patients. The mean age of the patients was 59.1 years (standard deviation ±16.2) for the cases, and 58.4 years (standard deviation ±15.2) for the controls.

Table 1 presents the description of the intrinsic and extrinsic factors for the occurrence of the event.

**Table 1:** Distribution of intrinsic and extrinsic risk factors for falls (n=358). Porto Alegre, RS, Brazil, 2013-2014

Risk factors		Case			Control			Total	
		(n=179)	%		(n=179)	%		(n=358)	%
Intrinsic factors:									
	Walking limitation	145	81.0		120	67.0		265	74.0
	Previous fall	80	44.6		54	30.1		134	37.4
	Disorientation/confusion	73	40.7		31	17.3		104	29.0
	Frequent urination	57	31.8		31	17.3		88	24.5
	Urinary/intestinal urgency	54	30.2		30	16.8		84	23.4
	Postoperative period	41	22.9		58	32.4		99	27.6
	Drowsiness	37	20.7		24	13.4		61	17.0
	Agitation	24	13.4		5	2.7		29	8.1
Extrinsic factors:									
	Length of stay (days)*	12 (05;20)			11 (05;17)			11 (5;18)	
	Absence of caregiver	116	64.8		73	40.7		189	52.7
	Absence of the nursing diagnosis Risk for Falls†	85	47.4		118	66.5		203	56.7
	Sedation therapy (within 72 hours)	81	45.3		62	34.6		143	39.9
	Benzodiazepines therapy (within 24 hours)	63	35.2		47	26.3		110	30.7

*Median (percentages 25%; 75%). †Nursing diagnosis - NANDA International(20).

Regarding the number of administered medications (last dose of the classes: benzodiazepines, opioids, barbiturates, antipsychotics, antidepressants, antihypertensives, laxatives, diuretics, antihistamines, anticonvulsants, and sedatives) within 72 hours, the median was equal to three, with 0 (zero) as a minimum and eight as a maximum. 

**Figure 2:**
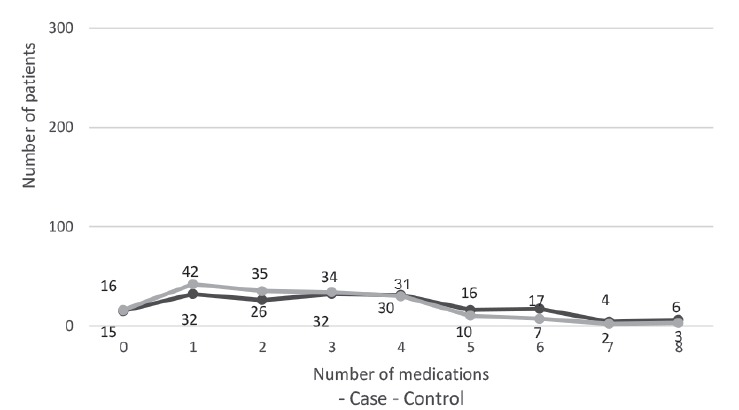
Number of medications administered prior the fall (n=358). Porto Alegre, RS, Brazil, 2013-2014

Figure 2 displays the distribution of the number of medications administered between cases and controls.

Table 2 presents the risk prediction for the investigated variables. 

**Table 2:** Results of the univariate logistic regression (n=358). Porto Alegre, RS, Brazil, 2013-2014

Variables	OR*	CI^†^ (95%)	p-value
Disorientation/confusion	4.45	[2.32 to 8.57]	<0.001
Walking limitation	3.62	[1.96 to 6.68]	<0.001
Absence of caregiver	0.42	[0.27 to 0.64]	<0.001
Absence of the nursing diagnosis Risk for Falls^‡^	2.43	[1.50 to 3.96]	<0.001
Urinary/intestinal urgency	2.56	[1.44 to 4.57]	0.001
Previous fall	2.11	[1.34 to 3.34]	0.001
Agitation	3.50	[1.41 to 8.67]	0.007
Frequent urination	2.46	[1.29 to 4.69]	0.006
Number of administered medications^§^	1.17	[1.41 to 1.31]	0.008
Length of stay (days)	1.06	[1.01 to 1.11]	0.01
Benzodiazepines therapy (within 24 hours)	1.78	[1.11 to 2.85]	0.01
Sedative therapy (within 72 hours)	1.92	[1.17 to 3.14]	0.01
Postoperative period	0.58	[0.34 to 0.96]	0.05
Drowsiness	1.87	[1.00 to 3.49]	0.05
Age (>60 years)	2.61	[0.58 to 11.79]	0.21

*Odds Ratio. †Confidence Interval. ‡Nursing diagnosis - NANDA International^20^. §Number of medications - Last dose of the classes: benzodiazepines, opioids, barbiturates, antipsychotics, antidepressants, antihypertensives, laxatives, diuretics, antihistamines, anticonvulsants, and sedatives administered within the 72 hours.

A multivariate logistic analysis was performed using the findings of the univariate analysis and the most important risk factors were identified for hospitalized adult patients (Table 3).

**Table 3:** Results of the multivariate logistic regression model with p<0.05 (n=358). Porto Alegre, RS, Brazil, 2013-2014

Variables	OR*	CI^†^ (95%)	p-value
Disorientation/confusion	4.25	[1.99 to 9.08]	<0.001
Frequent urination	4.50	[1.86 to 10.87]	0.001
Walking limitation	4.34	[2.05 to 9.14]	<0.001
Absence of caregiver	0.37	[0.22 to 0.63]	<0.001
Postoperative period	0.50	[0.26 to 0.94]	0.03
Number of medications administered prior the fall (within 72 hours)^‡^	1.20	[1.04 to 1.39]	0.01

*Odds Ratio. †Confidence Interval. ‡Number of medications - Last dose of the classes: benzodiazepines, opioids, barbiturates, antipsychotics, antidepressants, antihypertensives, laxatives, diuretics, antihistamines, anticonvulsants, and sedatives administered within the 72 hours.

## Discussion

This study presented the largest casuistry with a case-control design and falls as outcome in adult patients hospitalized in clinical and surgical units, and its findings reinforce the importance of intrinsic and extrinsic risk factors related to the neurological status of patients (disorientation/confusion), the alteration in urinary elimination (frequent urination), and the physical mobility (walking limitation). However, these findings differ from other studies^7^ ^-^ ^11^ ^,^ ^13^ ^-^ ^14^ ^,^ ^21^ ^)^ that aimed to identify risk factors for falls, as our study shows the relevance of the postoperative condition and extrinsic factors, such as absence of caregiver at the time of the fall, and number of medications administered before the occurrence of the event.

The risk factors identified in this study were: disorientation/confusion; frequent urination; walking limitation; absence of caregiver; postoperative period, and number of medications administered within 72 hours before the fall (last dose of the classes: benzodiazepines, opioids, barbiturates, antipsychotics, antidepressants, antihypertensives, laxatives, diuretics, antihistamines, anticonvulsants and sedatives). These risk factors prevail in elderly, and are in agreement with the epidemiological profile of the sample, i.e., mean age of 59.1 years (standard deviation ±16.2) for the cases and 58.4 years (standard deviation ±15.2) for controls. On the other hand, in this study, age greater than 60 years did not appear as a statistically significant variable.

The incidence of the event was higher in male patients (57%). However, there is no consensus in the literature on the association between sex and an increased risk for falls^22^ ^-^ ^24^. For this reason, this variable was one of the criteria adopted for the matching in this study.

The descriptive data presented in Table 1 showed that the length of hospital stay was one of the variables significantly related to the event. The median of the length of hospital stay was similar in both cases and controls, as this variable was investigated until the incidence of the fall. As it was a case-control study, no follow-up of these patients was performed after the event.

This has a direct relationship with a better clinical profile of these patients, what corroborates a lower number of patients in postoperative period in the sample (cases=22.9% and controls=32.4%). In general, in hospital clinical practice, non-surgical patients had a higher rate of falls when compared to surgical patients (considered in this study with a history of surgery(s) in the current hospitalization), as the former had a longer length of hospital stay, a higher incidence of comorbidities and a greater demand for health care^9^ ^,^ ^12^ ^,^ ^21^ ^-^ ^22^.

In this study, the postoperative period served as a predictor of the incidence of falls, although it did not show a greater significance level when compared with other factors. This is in agreement with the literature on this phenomenon^20^ ^,^ ^25^ ^-^ ^26^. The behavior of this variable could be interpreted as contrary, that means, it is known that different studies confirmed the postoperative period as an important risk factor for falls^16^ ^,^ ^18^ ^,^ ^27^. However, the complexity of non-surgical patients could have influenced the behavior of the predictive variables, such as the postoperative period.

Like the postoperative period, other variables showed a lower OR value (absence of caregiver at the time of the fall and the number of medications administered prior the event). This situation is explained by the Berkson’s Fallacy (individuals with two or more diseases create a different distribution of the exposure to the event)^28^, which may have influenced the pattern of the variables investigated. 

Among the continuous variables, in addition to a longer length of hospital stay, it is highlighted the number of medications administered (last dose of the classes: benzodiazepines, opioids, barbiturates, antipsychotics, antidepressants, antihypertensives, laxatives, diuretics, antihistamines, anticonvulsants, and sedatives) within 72 hours before the nursing evaluation and/or before the fall. This latter variable presented a median equal to three with a minimum of zero and maximum of eight medications.

A relevant element in this discussion is the polypharmacy use and its relationship with different and/or multiple comorbidities. Among the categorical variables related to medications, the use of sedatives within 72 hours and the use of benzodiazepines within 24 hours presented increased OR in the univariate regression. However, these findings were not the same in the multivariate regression, which found the number of medications administered before the event as a significant factor. 

The use of anticonvulsants medications and benzodiazepines was also investigated using the Hendrich II Fall Risk Model^29^. The administration of medications of different classes (tranquilizers/sedatives, diuretics, antihypertensives, antiparkinsonians, antidepressants and others) is also part of the Downton Fall Risk Index*,* which has been not fully tested and disseminated across studies^15^ ^,^ ^30^.

In an integrative review that aimed to find risk factors for falls, as this study, antidiabetic agents were found in only two observational studies^15^. Therefore, from the researchers’ point of view, there was not enough evidence to associate them with the outcome and they were not included in this study. 

In addition, when considering medications as predictors, the researchers point out that the association between different medications of the same class or the combination of different classes may produce or potentiate clinical conditions of hypotension, confusion, dizziness, attention deficit, drowsiness, and other. Furthermore, the researchers report that polypharmacy use should be supervised by health professionals, in order to identify factors that may contribute to the incidence of falls^31^ ^-^ ^32^.

Among these factors, the categorical variables with a greater weight were disorientation/confusion, frequent urination and walking limitation when compared to the others.

A research evaluating the risk for falls in adult patients admitted to clinical and surgical units in a teaching hospital in Southern Brazil, of a cohort of 831 patients, found that 19 patients suffered fall during the data collection period, and 63.2% (n=12) of these patients have already had the incidence of fall in the previous three months^33^. It is known that among the factors that independently correlate with an increased risk of falls are walking limitation, frequent urination and change in mental status (e.g. disorientation/confusion and drowsiness)^15^ ^,^ ^33^ ^-^ ^34^. These items are evaluated by the most relevant predictive models^29^ ^-^ ^30^.

Regarding the alteration in urinary and/or intestinal eliminations, the variable frequent urination was found as a predictive factor. This variable is so important that the Hendrich II Fall Risk Model^29^ includes the presence of urinary and/or intestinal alteration between its evaluated items. The same is true of the Risk Assessment Tool in Falling Elderly Inpatients (STRATIFY)^35^, which evaluates the frequency that patients go to the toilet. One explanation is that a more frequent need of to urinate is related to a greater need to go to the toilette, which exposes the patients to a greater risk of falling^24^ ^,^ ^33^ ^,^ ^36^. Environmental risk factors were not evaluated in the controls due to the limitations inherent to the case-control studies, in which patients are evaluated before the incidence of the event.

The results in Table 2 showed that 35.2% (n=63) of the cases were followed by a caregiver at the time of the fall and 59.3% (n=106) of the controls. In some situations, the family members were present, but were not able to interfere in the event, for example, when they were sleeping or when they were walking at the side of the patient, but were unable to hold them. Perhaps, in this study, if the incidence of the event was also considered as the absence of caregiver at the time of the fall, the behavior of this variable could be different.

This has a direct relationship with safety culture issues, when family members frequently assume tasks that should be under the responsibility of the nursing team, such as assisting with the bathing and/or in case of transfer. We emphasize that during the night the patients usually do not ask for the assistance of the nursing team, and many times, they hesitate in asking the assistance of their caregiver because they are sleeping^23^ ^,^ ^36^.

Nonetheless, these situations reflect the reality of many health institutions, where there is a push in stimulating the participation of family members in patient care^36^ ^-^ ^37^, in addition to an increased number of patients per nurse and an increased demand at work^7^ ^,^ ^11^. All these explanations are closely related to the dichotomous variable absence of caregiver and understanding of the reasons for possibilities care obtained by conditional logistic regression.

In this study, around 45% of patients with fall did not have the nursing diagnosis Risk for Falls, in both samples. The variable was analyzed just as a dichotomy, with or without the presence of the outcome. In contrast, a prevalence study identified that 86.2% (n=69) of the patients had the nursing diagnosis Risk for Falls during the admission. We highlight that in this just mentioned study, the diagnosis was raised by the researchers, which is not a reflection of the clinical practice reality^25^.

In another study, using clinical practice and carried out in the same institution of this study, a prevalence of 4% was identified for the use of the nursing diagnosis Risk for Falls in a sample of 174 patients in clinical and surgical units. Data were collected in 2011 from the computerized system and electronic chart, specifically from the nursing order sets^20^.

The authors point out that this finding may be related to the moment experienced at that time, when the institution was in the initial process for the international hospital quality accreditation^20^, what was achieved in 2013. It was also emphasized the importance of considering that nurses were not identified as risk factors and, consequently, an association could not be established. This reinforces the need and the importance of knowing the significant risk factors for the incidence of the event, as well as of adopting an accurate predictive instrument in the clinical practice. 

In nursing practice, a precise identification of predictive factors (risk factors) for the incidence of falls facilitates clinical reasoning of the nurses. Thus, this also helps in the assessment of the nursing diagnosis Risk for Falls and in the accomplishment of a care plan focused on preventive measures and patient safety. 

It is highlighted as limitations of this study that it was carried out in a single center, with secondary use of the data from the electronic chart and from the instrument of notification of falls of the institution. In addition to these, there is a risk of bias inherent of retrospective studies, for example, when patients were asked to remember the information prior to the event, which means that the evaluation was biased by the memory of patients.

## Conclusions

The risk factors for falls disorientation/confusion, frequent urination, walking limitation, absence of caregiver, postoperative period and number of medications administered within 72 hours before the fall (last dose of the classes: benzodiazepines, opioids, barbiturates, antipsychotics, antidepressants, antihypertensives, laxatives, diuretics, antihistamines, anticonvulsants and sedatives) support the individual clinical decision. This is true specifically to nurses, who need a better evidence to reliably identify the patient’s real risk of falling and to implement the best preventive interventions for the event. 

This study presented the largest casuistry with a case-control design and fall(s) as outcome, in adult patients hospitalized in clinical and surgical units. Its findings emphasize the importance of intrinsic risk factors and show that extrinsic factors, specifically those related to processes, such as absence of caregiver at the time of the event, contribute significantly to the incidence of the outcome.

In education, the understanding of predictors for falls facilitates the critical thinking and clinical judgment of the student, specifically in the identification of patients with moderate or high risk for falls. In addition, they can contribute to the understanding of more robust research designs.

In research, the support of a statistical and epidemiological reference can stimulate the development of future research and the establishment of new hypothesis, whose main outcome is patient safety.
